# Dynamics of Broadband Lasing Cascade from a Single Dot-in-well InGaAs Microdisk

**DOI:** 10.1038/s41598-019-41307-w

**Published:** 2019-04-04

**Authors:** Vadim Talalaev, Natalia Kryzhanovskaya, Jens W. Tomm, Viktoriia Rutckaia, Joerg Schilling, Alexey Zhukov

**Affiliations:** 1 Martin-Luther University, Centre for Innovation Competence SiLi-nano, Halle, 06120 Germany; 2 Saint-Petersburg Academic University, St. Petersburg, 194021 Russia; 3 Max Born Institute for Nonlinear Optics and Short Pulse Spectroscopy, Berlin, 12489 Germany

## Abstract

The development of a fast semiconductor laser is required for the realization of next-generation telecommunication applications. Since lasers operating on quantum dot ground state transitions exhibit only limited gain due to the saturation effect, we investigate lasing from excited states and compare its corresponding static and dynamic behavior to the one from the ground state. InAs quantum dots (QDs) grown in dot-in-well (DWELL) structures allowed to obtain light emission from ground and three excited states in a spectral range of 1.0–1.3 *μ*m. This emission was coupled to whispering gallery modes (WGMs) of a 6 *μ*m microdisk resonator and studied at room temperature by steady-state and time-resolved micro-photoluminescence. We demonstrate a cascade development of lasing arising from the ladder of quantum dot states, and compare the lasing behavior of ground and excited state emission. While the lasing threshold is being increased from the ground state to the highest excited state, the dynamic behavior is improved: turn-on times and lifetimes of WGMs become shorter paving the way towards high frequency direct driven microlasers.

## Introduction

The current interest in modern nanophotonics has motivated numerous studies of microcavity lasers based on microdisks and microrings^1^. Cavities with rotational symmetry support high-quality factor (Q) whispering gallery modes (WGMs) and exhibit advantages such as low threshold, small foot-print, small mode volume, and control of in-plane emission wavelength by the cavity diameter^2,3^. In WGMs, the mode fields are concentrated within several hundreds of nanometers from the disk’s edge and allow for efficient coupling with emitters placed there. Self-organized InGaAs quantum dots (QDs) formed in a GaAs matrix and emitting in the near infrared (NIR) region are very attractive for high-speed telecom applications^4,5^. Such QDs cause strong carrier localization that provides high temperature stability, elevated operating temperatures, and high signal-to-noise ratios^6^. This is of special interest for nanophotonic applications to avoid increased carrier recombination at the surfaces of small resonators. However, the optical gain from the ground state (GS) transition in QDs is limited due to the finite number of carriers in the QD ground state (two electrons on an s-like level). The problem of gain saturation can be overcome by the employment of excited states (ES). While the lasing at WGMs from GS transitions in QD cavities were theoretically and experimentally thoroughly investigated^7–12^, the lasing from ES in a QD laser microcavity has not yet been well studied. This is partly due to the fact that it is quite difficult to obtain large defect-free QDs with a well-defined set (ladder) of optical transitions.

Lasing dynamics is a decisive characteristic of cavity lasers for telecom applications and especially the turn-on dynamics is important for high data rate transmission^13^. Nevertheless, up to now the dynamics of WGMs in microdisks of small diameters was not sufficiently investigated. The temporal response of QD microdisk lasers characterized by a turn-on delay time and the radiative lifetime is hardly known. Gain-switched QD lasers show a fast dynamical response, which is characterized by the turn-on delay time, as well as damped relaxation oscillations^14^. Nevertheless, the explanation of the underlying dynamical processes is still under discussion.

In this work, we investigate lasing in microdisks, 6 *μ*m in diameter, with a dot-in-well (DWELL) structure as active medium. The direct deposition of InGaAs quantum wells (QWs) on InAs QDs allowed growing large-scale defect-free QDs with a set of states ranging from GS (transition at 1.3 *μ*m) up to the third excited state (ES3) with an emission peak near 1 *μ*m. Thus, we achieve two goals simultaneously: a lasing wavelength of 1.3 *μ*m that is relevant for telecom and the presence of several excited states in the QDs, which allows us to make a direct comparison of lasing on different QD states. Applying steady-state and time-resolved micro-photoluminescence measurements we investigate the spectral distribution as well as the dynamic behavior of the stimulated emission in this system. We present a complementary study on the kinetics of a complex QD band structure, and for practical applications, i.e. telecom lasers on excited states, the entire scheme can be shifted to lower energies by varying the QD size.

The paper is organized as follows: after the description of methods used in this study, we present steady-state and time-resolved luminescence of the unprocessed epitaxial substrate, which leads to the cascade model of the carrier relaxation in the QDs. Then we show the coupling of the QD emission to the WGMs of a microdisk fabricated on the substrate. Next, we present a static lasing in the microdisk, which allows obtaining Q-factors and threshold powers for lasing on different QD optical transitions. And in the last section, we discuss the lasing dynamics of the microdisk and identify the maximum modulation frequency and damping factor of the laser.

## Methods

### Sample preparation

Epitaxial structures were grown by molecular beam epitaxy on a semi-insulating GaAs (100) substrate. The active region comprised five layers of DWELL (In_0.15_Ga_0.85_As QWs deposited directly on InAs QDs) separated by 35 nm GaAs spacers. This active area was surrounded on both sides by 20 nm thick Al_0.3_Ga_0.7_As barriers and placed in the middle of a 330 nm thick GaAs waveguide layer grown on top of a 450 nm Al_0.98_Ga_0.02_As cladding layer.

**Figure 1:**
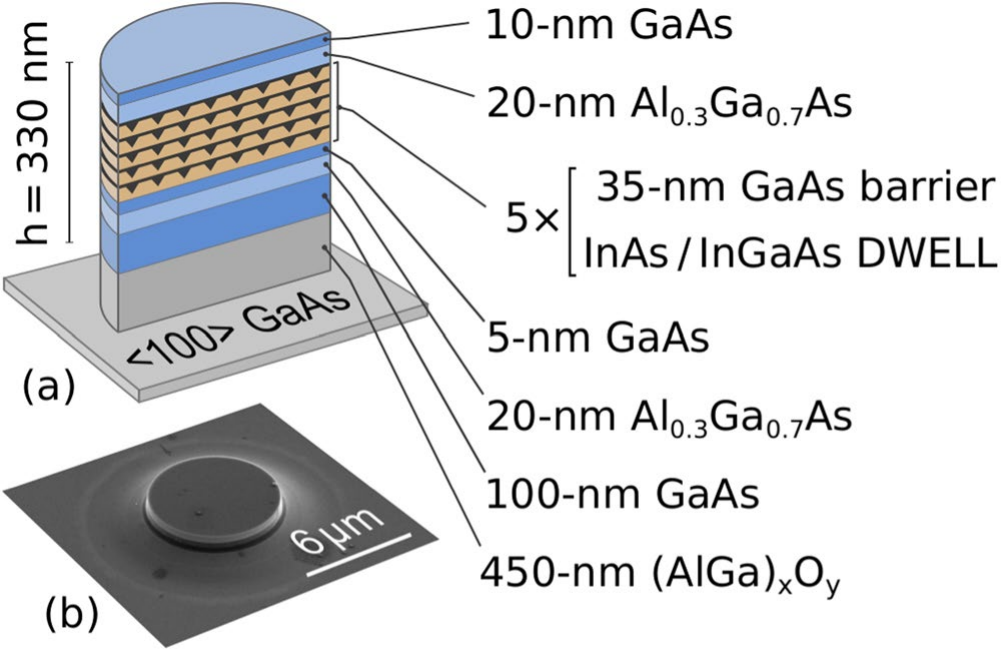
(**a**) Schematic cross-section of the microdisk fabricated on the epitaxial structure with InGaAs DWELL. (**b**) SEM image of the fabricated 6 *μ*m diameter microdisk.

Microdisks of a 6 *μ*m diameter were fabricated using photolithography and reactive ion etching. After that, the 450 nm thick Al_0.98_Ga_0.02_As layer was transformed into (AlGa)_x_O_y_ layer by a selective oxidation process to ensure the optical confinement from the substrate side. A schematic cross-section (a) and scanning electron microscope image of the grown structure (b) are shown in Fig. 1. The lateral density of QDs in each layer of 3.5 × 10^10^ cm^−2^ has been determined from plan-view TEM data (not shown). Thus, the total number of QDs in the disk is N_0_ = 5 × 10^4^.

### Micro-photoluminescence

The single microdisks under investigation were characterized by micro-photoluminescence (*μ*PL) at ambient temperature. To prevent heating, the sample was attached to a Peltier element. An exciting laser spot was focused on the microdisk’s surface by a 100× microscope objective (Mitutoyo, M Plan APO NIR HR) with a numerical aperture NA = 0.7. Both steady-state PL (CW*μ*PL) for light-in/light-out measurements and time-resolved PL (TR*μ*PL) for the study of the dynamics were carried out in a back-scattering geometry, i.e. emitted light was collected from the disk plane by the same objective (normal incidence) and directed by mirror optics into a grating monochromator.

CW*μ*PL was excited by the second harmonic of a cw-operating YAG:Nd laser with a wavelength of 532 nm. The static lasing behavior of the WGMs (light-in/light-out dependence) was investigated by measuring the PL spectra as a function of pump power. For this, a set of neutral density filters with a 0.1-optical density step was used to adjust the excitation power. We consider *light-in power on disk*, i.e. power brought to the active DWELL area without taking into account reflection losses and internal losses in the disk. Collected PL light was focused on the entrance slit of a 0.5-m grating monochromator (Acton Research SP-2500) and detected by a liquid-nitrogen cooled NIR-CCD with a linear InGaAs photodiode array (Princeton Instruments OMA-V/LN 1024) with a cut-off wavelength of 1.7 *μ*m. The grating (1200 grooves/mm) and entrance slit width (25 *μ*m) combined with the CCD dispersion (25 pm/pixel) provided a spectral resolution of 45 pm.

TR*μ*PL was excited by a 775-nm fundamental emission line from a mode-locked Ti:Sapphire laser (Spectra-Physics “Tsunami”, repetition rate 80 MHz, pulse width 100 fs). The resulting PL was dispersed by a 0.3-m grating monochromator (Acton Research SP-2300) and recorded by a synchroscan streak-camera (Hamamatsu C5680) with NIR-enhanced S1 photocathode. The cathode’s spectral response in the NIR range from 1000 to 1300 nm is characterized by a drop in sensitivity by two orders of magnitude. The spectral resolution is about 1 nm at the central wavelength (1.15 *μ*m). The temporal resolution is better than 10 ps. An acceptable signal-to-noise ratio of the PL signal was achieved at the time-averaged light-in power of 50 *μ*W in the analog integration acquisition mode of the streak camera. This corresponds to 0.6 pJ per pulse, which creates about 2 × 10^6^ electron-hole pairs. The 100× objective focuses the excitation light on a spot of ∼1 *μ*m diameter on the sample. The penetration depth of the excitation pulse (780 nm) in the GaAs is ∼1 *μ*m. As the diffusion length of electrons in lightly doped GaAs (up to 20 *μ*m^15^) exceeds the disk size, a quasi-homogeneous non-equilibrium carrier concentration 2 × 10^17^ cm^−3^ in the microdisk can be assumed, which amounts to about 10^6^ electron-hole pairs in the active area. This is equivalent to 20 electron-hole pairs per QD.

### WGM modeling

Microdisk WGMs were modeled using the finite-element method by solver COMSOL multiphysics (eigenfrequency analysis) utilizing azimuthal symmetry^16^. The active area was set as a homogeneous GaAs medium with optical constants taken from ref.^17^. The (AlGa)_x_O_y_ pedestal was modeled as a lossless homogeneous medium with the refractive index of 1.6. The microdisk and the pedestal were surrounded by air.

## Results and Discussion

### PL from as-grown DWELL sample

**Figure 2:**
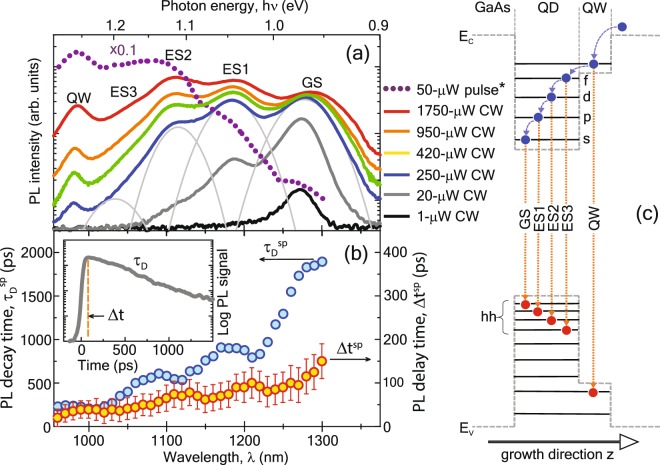
(**a**) PL spectra of as-grown DWELL structure under different pumping: solid lines – CW-excitation; dotted line – pulse excitation (*time-averaged light-in power of 50 *μ*W for a 100-fs-pulse). Deconvolution of the 250 *μ*W spectrum (blue) into four Gaussians corresponding to different optical transitions is shown in light-gray solid lines. (**b**) The data of TR*μ*PL measurements obtained from the analysis of PL temporal profiles (inset shows such profile for 1150 nm central wavelength): PL decay time *τ*_D_ is obtained by mono-exponential fitting; PL delay time Δ*t* is defined as a delay of maximal PL response with respect to the excitation pulse. The superscript “sp” denotes that these times belong to the spontaneous emission of the QDs. (**c**) Schematic of the DWELL structure. Arrows indicate the relaxation pathways for electrons (blue) as well as the PL recombination transitions (orange).

Since there is no cavity present in the unstructured DWELL sample, lasing does not yet occur and the PL signal discussed in this section corresponds to the spontaneous emission from the QDs. CW*μ*PL spectra of the as-grown InGaAs DWELL structure for various light-in power on the disk are shown in Fig. 2a. One can see the broadening of the PL signal with the increase of the excitation power, and the presence of several maxima, which reflect quantum energy-levels of different states in the epitaxial structure (a schematic of the QD optical transitions is shown in Fig. 2c). For low power (P = 1 *μ*W), the PL response occurs close to 1.3 *μ*m corresponding to optical transitions between electron-hole ground states (GSs) of the QDs. The luminescence profile is related to the size distribution of the QDs and characterized by the full width at half maximum (FWHM) of 40 nm (30 meV). Increasing light-in power leads to the sequential filling of excited states ES1, ES2, and ES3 up to QW states.

Due to the inhomogeneous broadening of the levels, PL bands overlap and the total signal is spectrally “blurred”. In order to analyze the population of QD electron levels, measured PL spectra were deconvoluted by four Gaussians corresponding to optical transitions from different QD states. The capacity of QD electron states according to the simplified atomic model grows as 2(s)/6(p)/10(d)/14(f). In comparison, the integrated PL intensity ratio of Gaussians for 250 *μ*W light-in CW-power (Fig. 2a) is 2(GS)/2(ES1)/1(ES2)/0.2(ES3), while an analogous deconvolution for the 1750 *μ*W CW-power gives the intensity ratio 2/2.5/2/1.5. For the pulsed power, the ratio is 2/3/6/4. These ratios imply that even for the highest pump power the excited states are not completely filled, which agrees with the 20 electron-hole pairs per QD estimation made in Methods section.

We characterize the temporal behavior of the luminescence by PL decay time *τ*_D_ and PL delay time Δ*t* obtained from high-excitation TR*μ*PL and shown in Fig. 2b. Under high-excitation conditions, the channels of non-radiative defect-assisted recombination are saturated and, therefore, can be neglected. In this case, the temporal profile can be fitted by one exponent, and the PL decay time will be mainly governed by the radiative lifetime of the as-grown DWELL structure. Such fit was done for each 10-nm spectral region of interest. With the same step, the PL delay time Δ*t* was determined from the temporal profiles as an interval between the excitation peak (t = 0) and the maximum of the PL signal. Both characteristics show the slowing of the PL response for transitions between deeper QD levels. The radiative lifetime *τ*_D_ increases from 210 ps for ES3 to 1800 ps for GS transitions, and the delay time Δ*t* also grows from 20 ps to 130 ps. These observations can be explained with the help of the transition cascade in Fig. 2c. After excitation, a large part of the excited electron-hole pairs occupy conduction and valence band levels in the GaAs waveguide and the QW-region of the microdisk. They relax first into the highest QD-levels (e.g. the f-level in Fig. 2c), so that the occupation of these excited QD-levels rises first resulting in the quick onset of the observed high energy (short wavelength) ES3 radiative transition. However, this transition is relatively short-lived since the carriers can relax further to several lower lying QD-levels emptying the highest QD-level. The strong competition of these intersubband relaxation channels with the radiative ES3 transition results in the short ES3-decay time of (*τ*_D_ = 210 ps). Next the slightly lower lying QD-d-level is filled giving rise to the radiative ES2-transition, which noticeably starts after the onset of the ES3-transition. However, also for this level, the intersubband relaxation to the lower p- and s- QD-levels competes with the radiative ES2-transition leading to a further trickling down of the excited carriers. This cascade of intersubband transitions - subsequently filling and emptying the different QD-levels - results in the stepwise increase of the delay times for the transitions ES3, ES2, ES1, and GS. When the remaining carriers reach the GS the cascade comes to an end and only the low energy radiative GS-transition occurs. Since this does not compete anymore with intersubband relaxation channels the GS- level exhibits the largest lifetime (*τ*_D_ = 1800 ps).

### PL from a microdisk with embedded DWELL structure

**Figure 3:**
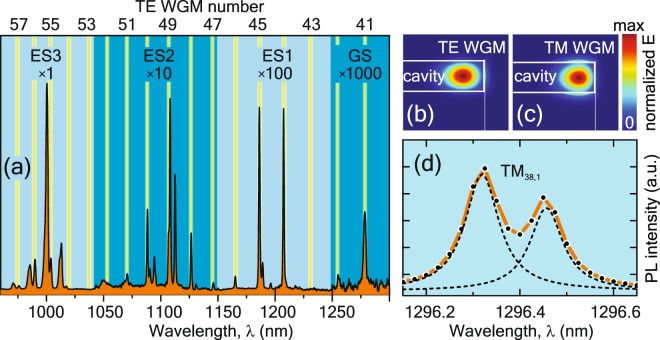
(**a**) Time-integrated *μ*PL spectrum of the disk. The integration of streak camera traces was done in the window of 0– 1600 ps for four separate spectral regions related to ES3, ES2, ES1 and GS Gaussians in Fig. 2a. The PL intensity was normalized individually for each region to take into account the drop of photocathode sensitivity (see Methods). Therefore, this figure serves not for the comparison of PL intensities, but for relating experimental peaks with fundamental WGM-resonances calculated by a finite-element method (yellow lines). Panels (**b**,**c**) – calculated spatial distribution of electric field magnitude E typical for TE and TM WGMs in a 6 *μ*m GaAs microdisk. (**d**) Zoom for CW*μ*PL spectrum with TM_38,1_ doublet and its deconvolution into two Lorentzians. Light-in power on disk is 200 *μ*W.

A typical time-integrated *μ*PL spectrum of a disk fabricated on the DWELL structure is shown in Fig. 3a. The transformation of the DWELL PL is evident: the broad bands turn into narrow lines due to the coupling of the QD emission to WGMs. Their spectral positions correspond to the wavelengths for which the resonance condition is satisfied, and a standing wave is formed by a defined number of internal reflections on the side wall of the microdisk. In order to identify dominant resonances in the PL spectrum, Fig. 3a also shows TE WGMs simulated by a finite-element method (vertical lines). Additional peaks correspond to higher order TE- or TM-modes. The mode profiles (normalized electric field) for TE and TM WGMs are shown in Fig. 3b,c and demonstrate the concentration of the WGM mode fields near the rim of the microdisks.

The far-field PL of microdisks exhibits the directional character of WGM emission^2,18^. Due to the radiation leakage through the side walls of the disk, the emission pattern of WGMs has a sharp maximum in the disk plane and no oblique components. This means, that in the back-scattering geometry used here, we should not detect WGM emission. However, Fig. 3 clearly shows the presence of the WGM-related peaks. We attribute this to the light scattering in a “non-ideal” microdisk, which enables emission along the oblique directions that can be collected by the high NA microscope objective. We performed PL measurements in two geometries: excitation at the 90°-angle (along the normal), collection at the same 90°-angle (back-scattering geometry); excitation like the previous one (90°), collection at the 7°-angle (with respect to the disk plane), and compared WGM intensities. It turned out that up to one-quarter of the maximum possible PL signal was collected in the case of back-scattering geometry. This confirms the presence of strong scattering and agrees with the data obtained in ref.^18^, where the angular dependence of WGM emission from the microdisks with GaAs QWs was measured.

The scattering was attributed to side-wall and surface roughness of the fabricated microdisks. Moreover, in our samples, the emitting medium consists of InAs QDs embedded in the GaAs-based waveguide. The QDs act as scattering centers due to the different refractive indices. The presence of scattering centers is disadvantageous for resonant characteristics, namely, it can lead to the decrease of a quality factor (Q-factor) and, hence, broadening of WGM lines^19^. For embedded nanoparticles (QDs), scattering also results in a splitting of WGM lines into doublets^20,21^. This splitting is caused by the lifting of the two-fold degeneracy of clockwise and counter-clockwise WGM waves^22,23^. The splitting of the TM_38.1_ WGM (1296 nm) into a doublet is demonstrated in Fig. 3d for CW-excitation. The doublet was deconvoluted into two Lorentzians exhibiting a spectral splitting of 130 pm and a FWHM of 90 pm for both components.

**Figure 4:**
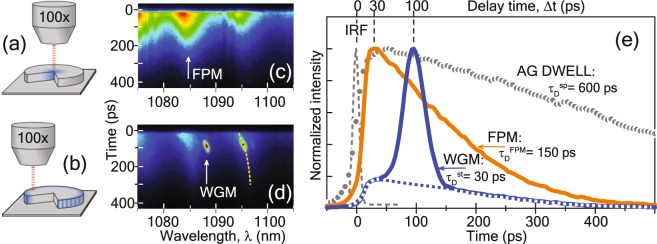
Excitation of the disk center (**a**) and disk periphery (**b**). Respectively, the detection of FPMs (**c**) and WGMs (**d**) on the streak camera panels. Color represents the intensity of the PL signal. (**e**) Temporal profiles of FPM and WGM TE_50,1_ denoted by the arrows on the detection panels (**c**,**d**). The profile of as-grown (AG) DWELL demonstrated here was measured at the wavelength that matches the WGM wavelength. Instrumental response function (IRF) indicates 10-ps time resolution and zero time for the excitation pulse.

Besides the splitting of the WGM modes, we have checked the spatial localization of the WGM modes by varying the position of the excitation/collection spot. The change of the TR*μ*PL spectrum by moving the 1 *μ*m wide excitation spot from the center to the periphery of the disk (Fig. 4a,b) is shown in Fig. 4c,d. When the excitation spot is placed at the disk center (4a), only Fabry-Perot modes (FPMs) are detected as broad and long oscillatory traces in the emission spectrum. The FPMs are formed by the radial propagation of the light between the disk center and the reflecting rim of the microdisk. For an excitation near the rim of the microdisks (4b) mainly the WGMs are excited (narrow short spots on the detection panel d) and the FPMs give only a minor contribution to the emission field at the periphery of the disks.

The resonances exhibit a “red” shift during the emission process, which appears in TR*μ*PL spectra as inclined traces (Fig. 4c,d). This behavior is attributed to the increase of refractive index Δn of the microdisk during the emission process. This can be explained by the decay of the non-equilibrium carrier concentration ΔN due to the recombination after the excitation pulse. The carrier-induced change of n is characterized by the Δn/ΔN ratio, which varies from −7 × 10^−21^ to −3 × 10^−22^ cm^3^ in literature^24^. As it follows from the WGM modeling, the overall shift of ∼1 nm measured for WGMs in Fig. 4d corresponds to the change of the refractive index of Δn ∼0.0015. This allows us to estimate the pulse-induced concentration of nonequilibrium carriers in the microdisk cavity ΔN = (2–50) × 10^17^ cm^−3^, which agrees with the value of 2 × 10^17^ cm^−3^ obtained from the absorbed power (see Methods section).

### CW lasing in DWELL microdisk

**Figure 5:**
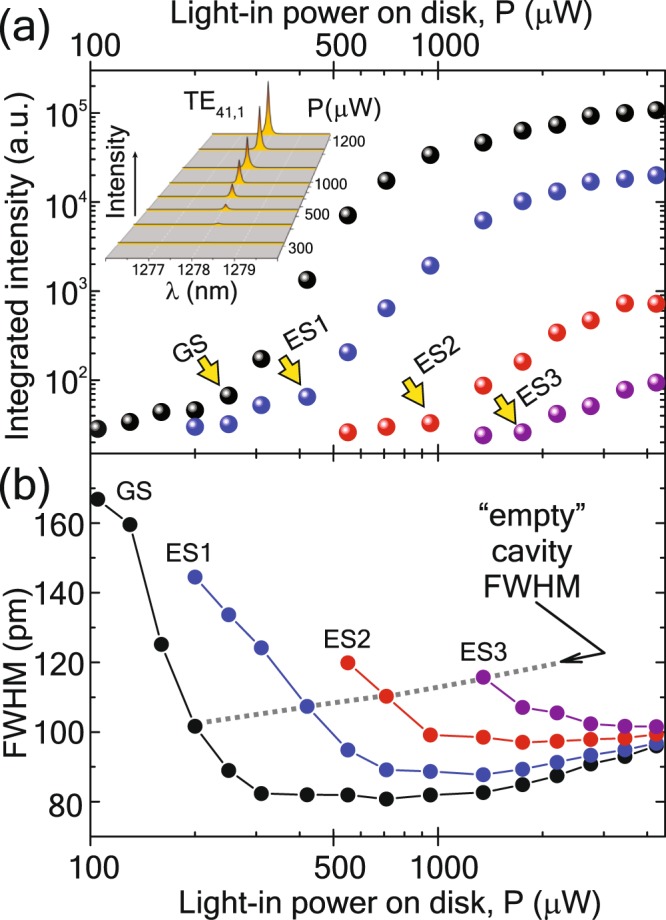
Processed lasing data for WGMs corresponding to different QD states. (**a**) Input-output (light-in/light-out) dependence. (**b**) FWHM versus light-in power on disk.

WGM cw lasing was investigated by measuring CW*μ*PL light-in/light-out power dependence of the PL. Inset in Fig. 5a shows such dependence for TE_41,1_ WGM. It exhibits a sudden increase in the peak intensity that indicates the achievement of the lasing threshold. Similar threshold behavior was also observed for other resonances. The following dominant WGMs were chosen to characterize the lasing from the different states of QDs: TE_41,1_ (1278 nm) for GS, TE_44,1_ (1207 nm) for ES1, TE_49,1_ (1108 nm) for ES2, and TE_55;1_ (1001 nm) for ES3. The WGM peaks were fitted by Lorentzians, and their integrated intensity is shown in Fig. 5a. The threshold power (Pth) was determined as a kink in the integrated intensity and is indicated with yellow arrows. One can see that excited states require higher excitation power to achieve lasing. The cascade development of lasing originates from the ladder of QD states. A comparison of the threshold values (Fig. 5a) with the power dependence of as-grown DWELL PL (see the last section) leads us to a conclusion that the lasing on the ES transitions does not require the complete filling of the corresponding QD states.

Along with the nonlinear increase in the emission intensity, a narrowing of the emission peaks occurs (Fig. 5b). The FWHM at the “empty cavity” limit was determined for peaks at an excitation intensity just one filter step (optical density = 0.1) below the lasing threshold. The FWHM-values, in this case, are used to determine the Q-factor of the cavity before the emission is governed by stimulated emission. Using the obtained Q-factors, Purcell factor was calculated as ${F}_{{\rm{p}}}=\frac{3}{4{\pi }^{2}}\frac{Q}{{V}_{{\rm{m}}}}{(\frac{\lambda }{{n}_{{\rm{eff}}}})}^{3}$, where *n*_eff_ is the effective refractive index (we took the constant value *n*_eff_ = 3.5 for GaAs). The mode Volume *V*_m_ was obtained from numerical modeling as the ratio of the total electric energy in the microdisk and the maximum value of the electric energy density ${V}_{m}=\frac{{\int }_{V}\varepsilon (\overrightarrow{r}){|\overrightarrow{E}(\overrightarrow{r})|}^{2}{d}^{3}r}{{\rm{\max }}(\varepsilon (\overrightarrow{r}){|\overrightarrow{E}(\overrightarrow{r})|}^{2})}$. The lasing characteristics of WGMs for different QD states are summarized in Table 1; the transient behavior of the luminescence is described below.

**Table 1:** CW lasing characteristics of WGMs in the disk cavity.

Transition	WGM	*λ* (nm)	Pth (*μ*W)	Δ*λ* (pm)	Q (×10^4^)	Fp	${{\boldsymbol{\tau }}}_{{\bf{D}}}^{{\bf{sp}}}$ (ps)	${{\boldsymbol{\tau }}}_{{\bf{D}}}^{{\bf{st}}}$ (ps)	Δ*t*^st^ (ps)
GS	TE_41,1_	1278	250	102	1.3	68	1800	125	370
ES1	TE_44,1_	1207	420	107	1.1	50	820	60	170
ES2	TE_49,1_	1108	950	110	1.0	34	550	30	100
ES3	TE_55,1_	1001	1750	116	0.9	22	210	10	20

The threshold power P_th_ is detected as a kink in the light-in/light-out dependence, full-width at half-maximum Δ*λ* is obtained from Lorentzian fitting, quality factors Q were calculated at the “empty” cavity condition – one step (optical density 0.1) below the laser threshold as *λ*/Δ*λ* The Purcell factor was obtained as ${F}_{{\rm{p}}}=\frac{3}{4{\pi }^{2}}\frac{Q}{{V}_{{\rm{m}}}}{(\frac{\lambda }{{n}_{{\rm{eff}}}})}^{3}$, spontaneous ${\tau }_{{\rm{D}}}^{{\rm{sp}}}$ and stimulated ${\tau }_{{\rm{D}}}^{{\rm{st}}}$ PL lifetimes were obtained from TR*μ*PL measurements of the unprocessed DWELL structure and microdisk, respectively. The delay time Δt^st^ was determined as the time difference between the excitation pulse and the maximum of the PL intensity.

### Lasing dynamics of WGMs

For TR*μ*PL measurements of microdisks, a 50 *μ*W (time-averaged) power pulsed excitation was used corresponding to 0.6 pJ; the temporal and spectral resolutions were 10 ps and 1 nm, respectively. A comparison with the CW spectra in Fig. 5a clarifies that these TR*μ*PL measurements were made above the lasing threshold.

#### WGM lifetime

**Figure 6:**
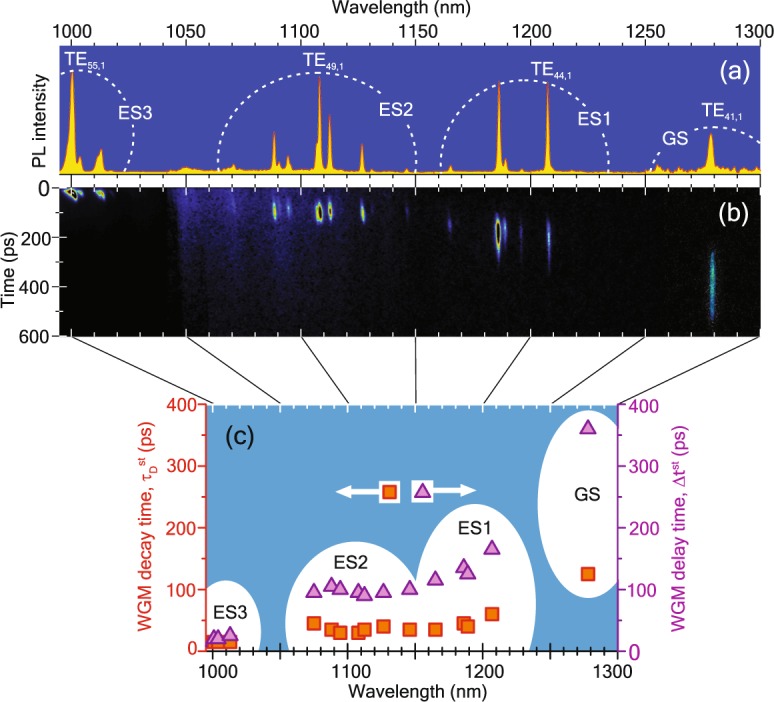
(**a**) Spectrally normalized time-integrated PL for the disk. (**b**) Original streak camera images used for time integration. (**c**) Dynamic data processed from panel (b): WGM radiative lifetime ${\tau }_{{\rm{D}}}^{{\rm{st}}}$ (squares) and turn-on delay time Δt^st^ (triangles), versus spectral position of WGM were defined like the *τ*_D_ = t_AG_ and *τ*_R_ in Fig. 2b.

Figure 4e compares the transient behavior of the PL from an as-grown DWELL structure and from the microdisk. The ES2 QD transition (*λ* = 1088 nm) in an as-grown DWELL-structure has a long lifetime of ${\tau }_{{\rm{D}}}^{{\rm{sp}}}$ = 600 ps (shown with gray circles). When the microdisk is excited at the center the luminescence curve at the same wavelength of 1088 nm exhibits a shorter spontaneous emission lifetime of ${\tau }_{{\rm{D}}}^{{\rm{FPM}}}$ = 150 ps (solid orange line). This is connected with the excitation of the FPM as discussed before. The PL decay profile of the TE_50,1_ WGM (blue solid line) is obtained when the excitation spot is moved to the rim of the disk. It shows a more complex profile which consists of the peaked WGM emission with ${\tau }_{{\rm{D}}}^{{\rm{st}}}$ = 30 ps, and the underlying rather flat FPM emission (blue dotted line). The onset of the WGM lasing is delayed relative to underlying the spontaneous FPM emission and appears 100 ps after the excitation pulse forming the steep peak on top of the FPM emission. Summarized TR*μ*PL data for WGMs corresponding to different QD optical transitions are collected in Table 1 and in Fig. 6c. The radiative lifetime ${\tau }_{{\rm{D}}}^{{\rm{st}}}$ for the WGM lasing emission (Fig. 6c) shows the same trend as the radiative lifetime ${\tau }_{{\rm{D}}}^{{\rm{sp}}}$ of the as-grown, unstructured DWELL-structures (Fig. 2b): the shortest lifetime for the highest excited state, and the longest lifetime for the ground state.

#### WGM delay time

The appearance of emission in the WGM as a response to the pulsed excitation arises after a delay time Δ*t*^st^ (see Fig. 6b,c). For selected modes, Δ*t*^st^ are listed in Table 1 . Often the lasing delay is related to the time that photons stay inside the cavity, which is known as *photon lifetime within cavity τ*_ph_. Using the second definition of Q-factor as the ratio of the stored photon energy in the cavity to the energy loss per photon cycle^25^, *τ*_ph_ can be expressed as ${\tau }_{{\rm{ph}}}=\frac{Q}{\omega }=\frac{1}{2\pi cn}\frac{{\lambda }^{2}}{{\rm{\Delta }}\lambda }$, where *ω* is the cyclic photon frequency, c is the speed of light. For all investigated wavelengths, *τ*_ph_ is about 2 ps, which agrees with the value reported in ref. ^26^ but does not differ much for the considered wavelengths. Since in our case the observed delay times are one or two orders of magnitude larger and also show a clear wavelength dependence, they have to be governed by another process. We attributed the observed Δ*t*^st^ to the time that the population inversion takes to build up to satisfy the lasing conditions. This can be understood with the help of the relaxation cascade from Fig. 2b. After the excitation, electrons relax from the continuum to the nearest (highest) QD level within 1–10 ps^27^, so that the population inversion can build up in this level and lasing starts at the ES3-transition having only a very short turn-on delay of Δ*t*^st^ = 20 ps. After further relaxation of the carriers down, the cascade population inversion is next reached for the ES2-transition whose turn-on delay is already 100 ps relative to the excitation time. After population inversion for the ES1-transition is reached, finally also population inversion and lasing occurs at the GS. Since this is the final state of the relaxation cascade all remaining carriers will finally end up there and the GS, therefore, offers the longest lasing pulse (${\tau }_{{\rm{D}}}^{{\rm{st}}}$ = 125 ps). The turn-on dynamics of QD lasers is important for fast data transmission systems. These results demonstrate that a fast dynamical response should rely on the emission from excited states (ES1–3). Charge carriers are collected first in these excited states leading to short turn-dynamics. Furthermore, their emission is quenched effectively by the carrier relaxation to lower lying QD-states resulting in a potentially high modulation speed. The drawback is the higher threshold power necessary to obtain population inversion and lasing in these states.

#### Damped relaxation oscillations of ES3 WGMs

**Figure 7:**
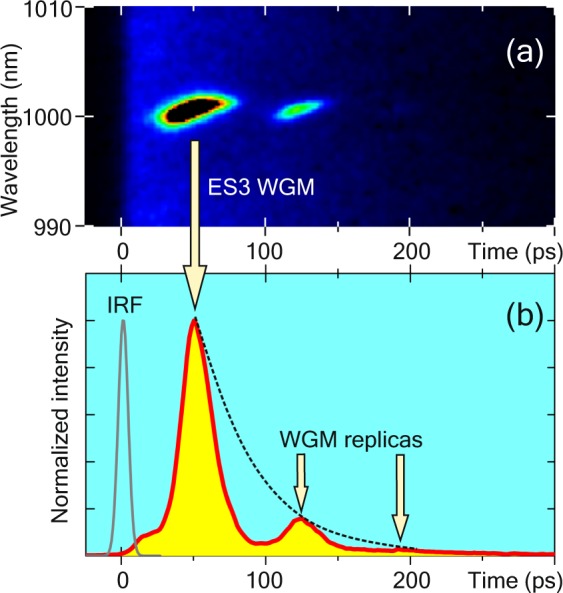
(**a**) Spectro-temporal traces of ES3 WGM affected by relaxation oscillations. (**b**) Transient profile of damped relaxation oscillations from panel (a). The 1-nm region of interest is centered at 1001 nm (TE_55,1_ WGM).

In the case of a slightly defocused excitation laser beam, we observed the oscillation of ES3-WGM-intensity in TR*μ*PL measurements, which is shown in Fig. 7. The damped relaxation oscillations are well known for electrically driven semiconductor lasers^28,29^ and occur due to the dynamic interaction between the photons and the carriers.

This modulation of the emission intensity can be explained by the out-of-phase correlation between the evolving photon and carrier densities^30^. An increasing number of photons in the cavity leads to the fast depletion of excited carriers by stimulated emission. When the population inversion is then run down, lasing stops and the number of photons decreases. However, subsequently the carrier density inside the QD can recover if there are enough carriers in the surrounding of the QDs leading to a new population inversion, and the cycle repeats resulting in an oscillation of the luminescence intensity. However, on average, emission intensity decays in an asymptotic manner as the number of charge carriers once excited is limited. These oscillations are important features of laser dynamics and limit the frequency with which a laser can be modulated; for high-frequency lasers, it is desired to have no damped relaxation oscillations.

We observed such damped relaxation oscillations with a period of 75 ps, i.e. relaxation frequency f_R_ = 13 GHz. A damping time of 40 ps was determined from the exponential fit of the intensity decay (dotted line in Fig. 7b). Following the formalism for the electrically driven lasers^28,31–33^, we introduce the following parameters: damping factor *γ* = 25 GHz (reciprocal of the decay period), modulation factor K, and maximal modulation bandwidth f_M_. These quantities relate to each other through equations $\gamma =K{f}_{{\rm{R}}}^{2}$ and ${f}_{{\rm{M}}}=\sqrt{1+\sqrt{2}}\cdot {f}_{{\rm{R}}}$. Substituting experimental values of f_R_ and *γ* in the equations above, we find K-factor and maximal modulation frequency: K = 150 ps and f_M_ = 20 GHz.

## Conclusion

A comparison of the lasing behavior of the whispering gallery modes on the ground and excited states of quantum dots in dot-in-well (DWELL) structure covering a spectral range of 1.0–1.3 *μ*m was carried out. Optically pumped single microdisks with a diameter of 6 *μ*m were measured at room temperature by steady-state and time-resolved micro-photoluminescence. Due to the large sizes of the investigated QDs, several higher QD-states exist. This has a crucial impact on the observed emission spectra and dynamics. Investigating the static and dynamic behavior of the lasing from GS and three ESs, we demonstrated a cascade nature of the excitation relaxation. We show that more power is required to reach the lasing threshold for the higher QD states deteriorating the cw characteristics of the lasing from these excited states. In contrast, the dynamic behavior is improved from GS towards ES3: turn-on times and lifetimes of WGMs become shorter resulting in accelerated lasing dynamics from QD excited states.

## Data Availability

All data generated or analyzed during this study are available upon a request.
